# 200. Rapid Initiation of Hepatitis C Treatment in Hospitalized Veterans Using a Real-Time Clinical Dashboard

**DOI:** 10.1093/ofid/ofaf695.072

**Published:** 2026-01-11

**Authors:** Edwin Wilbur Woodhouse, David Jacob, Karine Rozenberg-Ben-Dror, Colleen Boatright, Rachel B Britt, Christopher W Woods, David Ross, Susanna Naggie

**Affiliations:** Duke University Medical Center, Durham, NC; Central Texas VA, Temple, Texas; Veterans Administration, Woodland Hills, California; Veterans Health Administration, Durham, North Carolina; Veterans Health Administration, Durham, North Carolina; Duke University, Durham, North Carolina; Office of Specialty Care Services, Veterans Health Administration, Washington D.C., District of Columbia; DCRI/ School of Medicine, Durham, NC

## Abstract

**Background:**

Over the last decade, the Veterans Health Administration (VHA) has treated and cured over 125,000 veterans with chronic hepatitis C virus infection (HCV) with direct-acting antivirals (DAAs). However, over 21,000 veterans with HCV in VHA care remain untreated due to intractable patient- and system-level barriers. Because these hard-to-treat veterans may not reliably engage with care in the outpatient setting, the usual site of HCV treatment, we used an electronic population health clinical dashboard to facilitate HCV treatment initiation in the inpatient setting.

**Table 1:**
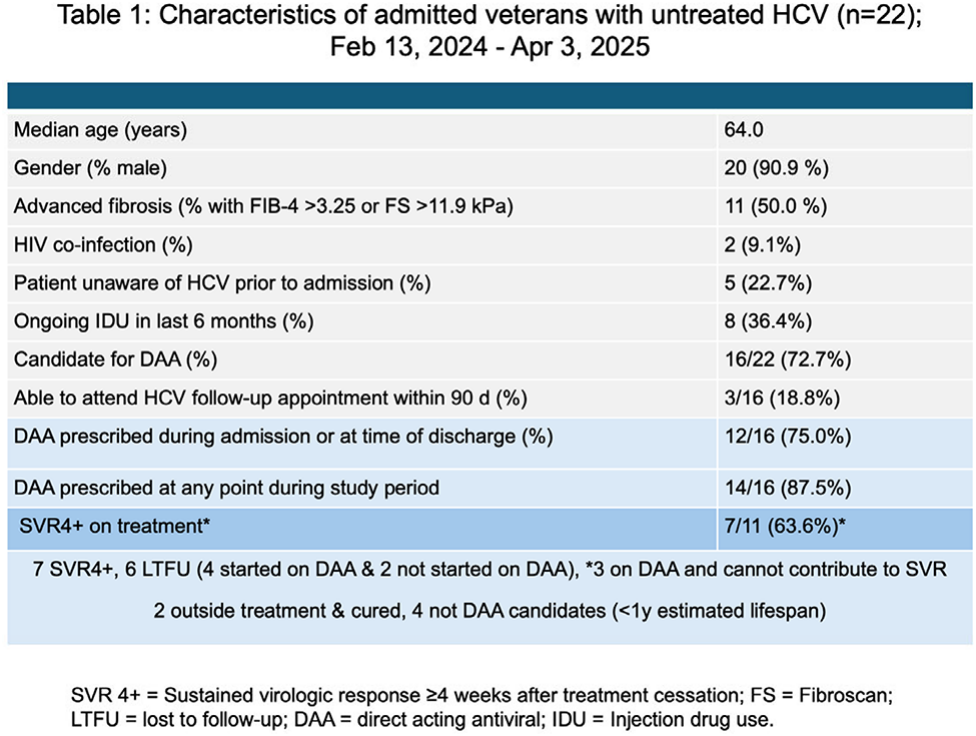
Characteristics of admitted veterans with untreated HCV (n=22); Feb 13, 2024 - Apr 3, 2025 Age, demographics, rate of DAA access, and SVR data for admitted veterans with HCV at Durham VAMC.

**Figure 1:**
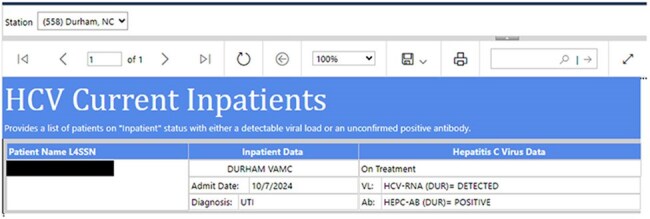
Example dashboard alert for admitted patients with hepatitis C Example dashboard alert automatically emailed to HCV clinicians when a veteran with untreated HCV is admitted to Durham VAMC.

**Methods:**

We used VHA’s National HCV Dashboard to alert an HCV clinician at the Durham Veterans Affairs Medical Center (DVAMC) via email when a veteran with active HCV infection was hospitalized (Fig. 1). The clinician evaluated the veteran for HCV treatment, and if clinically appropriate, offered initiation of DAA therapy during hospitalization or at the time of discharge (Fig. 2). We leveraged existing facility-level HCV infrastructure for follow-up and treatment monitoring.

**Figure 2:**
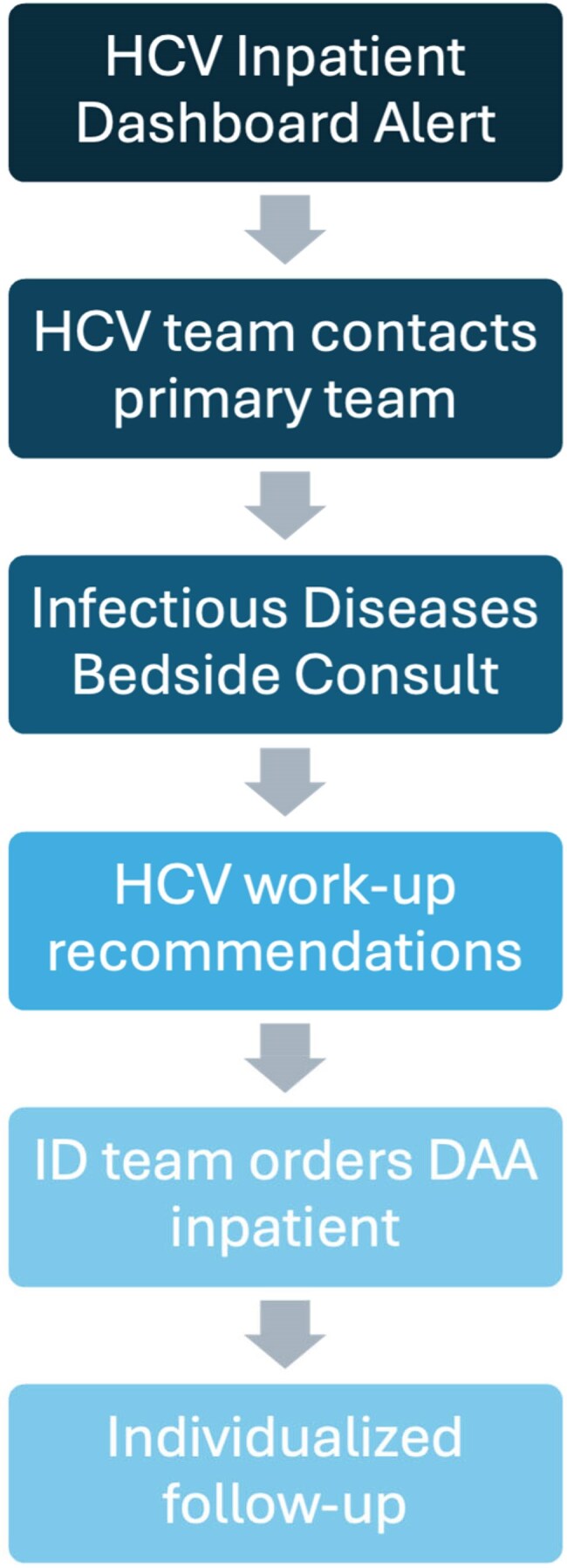
Inpatient HCV Workflow Process workflow for HCV treatment in admitted veterans at Durham VAMC

**Results:**

Twenty-two veterans with HCV, defined as prior HCV viremia without subsequent negative viral load, were admitted to the DVAMC between Feb 13, 2024 – Apr 3, 2025, of whom 16 were determined to be treatment candidates (Table 1). Of these, 14 (87.5%) were started on DAA therapy; 12 during admission or at time of discharge, and two in the outpatient setting >90 days after discharge. All 16 had follow-up visits scheduled, but only 3 (18.8%) were able to come to an appointment within 90 days after discharge. At time of analysis, 6 of the 16 (37.5%) were lost to follow-up, 3 (18.8%) were actively on DAA, and 7 (43.8%) achieved SVR ≥4 weeks after cessation of therapy. No virologic failures were seen despite several early DAA discontinuations (not related to adverse events).

**Conclusion:**

Inpatient evaluation and treatment initiation for HCV is feasible and comparably effective to an opportunistic test-and-treat intervention trial outside of the United States. This is the first reported use of a clinical dashboard to rapidly identify and treat inpatients with HCV. This approach could be used in other healthcare organizations to facilitate rapid HCV care and eliminate HCV as a public health threat.

**Disclosures:**

Susanna Naggie, MD/MHS, BMS/PRA: Event Adjudication Committee|Clinical Infectious Diseases: Deputy Editor|Vir Biotechnologies: Stocks/Bonds (Public Company)

